# Economic Evaluation of Sacituzumab Govitecan for the Treatment of Metastatic Triple-Negative Breast Cancer in China and the US

**DOI:** 10.3389/fonc.2021.734594

**Published:** 2021-10-28

**Authors:** Jigang Chen, Mingyang Han, Aihua Liu, Bo Shi

**Affiliations:** ^1^ Beijing Neurosurgical Institute, Capital Medical University, Beijing, China; ^2^ Department of Interventional Neuroradiology, Beijing Tiantan Hospital, Capital Medical University, Beijing, China; ^3^ Department of Neurosurgery, The Third Xiangya Hospital, Central South University, Changsha, China; ^4^ Department of Breast Surgery, People’s Hospital of Qinghai Province, Xining, China

**Keywords:** economic evaluation, Sacituzumab Govitecan, breast cancer, China, US

## Abstract

**Background:**

The effectiveness of Sacituzumab Govitecan (SG) for metastatic triple-negative breast cancer (mTNBC) has been demonstrated. We aimed to evaluate its cost-effectiveness on mTNBC from the Chinese and United States (US) perspective.

**Methods:**

A partitioned survival model was developed to compare the cost and effectiveness of SG *versus* single-agent chemotherapy based on clinical data from the ASCENT phase 3 randomized trial. Cost and utility data were obtained from the literature. The incremental cost-effectiveness ratio (ICER) was measured, and one-way and probabilistic sensitivity analyses (PSA) were performed to observe model stability. A Markov model was constructed to validate the results.

**Results:**

In China, SG yielded an additional 0.35 quality-adjusted life-year (QALY) at an additional cost of Chinese Renminbi ¥2257842. The ICER was ¥6375856 ($924037)/QALY. In the US, SG yielded the same additional QALY at an extra cost of $175393 and the ICER was $494479/QALY. Similar results were obtained from the Markov model. One-way sensitivity analyses showed that SG price had the greatest impact on the ICER. PSA showed the probability of SG to be cost-effective when compared with chemotherapy was zero at the current willing-to-pay threshold of ¥217341/QALY and $150000/QALY in China and the US, respectively. The probability of cost-effectiveness of SG would approximate 50% if its price was reduced to ¥10.44/mg in China and $3.65/mg in the US.

**Conclusion:**

SG is unlikely to be a cost-effective treatment of mTNBC at the current price both in China and the US.

## Introduction

Breast cancer is the most commonly diagnosed malignant tumor among females and also the leading cause of cancer death. Worldwide, there were about 2.1 million newly diagnosed female breast cancer cases in 2018 (1). Triple-negative breast cancer (TNBC), defined by a lack of estrogen receptor, progesterone receptor, and human epidermal growth factor receptor 2, constitutes about 15-20% among all breast cancers and its treatment is challenging due to high proliferation and frequent metastasis (2). Patients with metastatic TNBC (mTNBC) have poor survival outcomes (3). Nowadays, single-agent chemotherapy remains the standard of care for patients with mTNBC though it is associated with low response rates and short progression-free survival (4, 5).

Sacituzumab govitecan (SG) is an antibody-drug conjugate in which SN-38 (the irinotecan active metabolite), a topoisomerase I inhibitor, is coupled to the humanized antitrophoblast cell-surface antigen 2 monoclonal antibody hRS7 IgG1κ through the cleavable CL2A linker (6). Intravenous SG received accelerated approval from the Food and Drug Administration (FDA) of the United States (US) on 22 April 2020 for the treatment of mTNBC (7) and the FDA granted regular approval to SG on 7 April 2021 (8). These approvals were based on the results of a phase I/II trial (NCT01631552) (9) and a confirmatory phase III trial (NCT02574455) (10). In this randomized, phase III trial (ASCENT trial), SG was compared with single-agent chemotherapy for the treatment of patients with relapsed or refractory mTNBC and without brain metastases. The results revealed that SG notably prolonged median progression-free survival (PFS) [5.6 months *vs* 1.7 months; Hazard ratio (HR) for progression or death, 0.41; 95% confidence interval (CI), 0.32 to 0.52; P<0.001] and overall survival (OS) (12.1 months *vs* 6.7 months; HR for death, 0.48; 95% confidence interval (CI), 0.38 to 0.59; P<0.001) in comparison with single-agent chemotherapy.

SG has undoubtedly provided a new option for treating patients with mTNBC. However, SG treatment was associated with significantly higher costs, which might limit its access in many countries (11). There are currently no economic evaluations of SG for its use in the treatment of mTNBC. Taking cost-effectiveness into considerations in healthcare decisions is crucial for clinicians and policy-makers to allocate limited healthcare resources. Herein, we evaluated the cost-effectiveness of SG *versus* single-agent chemotherapy for mTNBC from the Chinese healthcare system and US healthcare payer perspective.

## Methods

### Patients and Intervention

This economic evaluation study was based on a literature review and modeling techniques, and it was deemed exempt from institutional review board approval because no real human participants were involved. The study was conducted according to the Consolidated Health Economic Evaluation Reporting Standards (CHEERS) reporting guideline (**Supplemental Table 1**) (12).

The target patient population was as same as that from the ASCENT trial. Included patients were adults (≥18 years of age) who had mTNBC that was relapsed or refractory to two or more previous standard chemotherapy regimens for unresectable, locally advanced, or metastatic disease. Patients with brain metastases were excluded. The median age of the patients was 56 years (8).

Included patients received either SG (10 mg per kilogram of body weight intravenously on days 1 and 8 of each 21-day cycle) or single-agent chemotherapy including eribulin (1.4 mg per square meter of body-surface area, intravenously on days 1 and 8 of a 21-day cycle, vinorelbine (25 mg per square meter intravenously on day 1 weekly), capecitabine (1000 to 1250 mg per square meter orally twice daily on days 1 to 14 of a 21-day cycle), or gemcitabine (800 to 1200 mg per square meter intravenously on days 1, 8, and 15 of a 28-day cycle). Chemotherapy was determined by the physician before randomization from one of these four single-agent treatments. Treatment would continue until disease progression or death.

### Model Construction

A partitioned survival (PS) model was constructed using TreeAge Pro 2020 (TreeAge, Williamstown, MA) to compare costs and clinical outcomes associated with SG *vs* chemotherapy for treatment of mTNBC. This model is frequently used in oncology modeling. In the PS model, the proportion of patients in different health states at different time points was derived from PFS (Progression-free survival) and OS (Overall survival) curves directly (13). Three different health states were included, which were progression-free disease state (PFD), progressed disease state (PD), and death. The proportion of patients in the PFD was obtained directly from the PFS curve while the proportion of patients in the death state was derived by 1 minus the OS curve at each time point. With regard to the PD, its proportion was derived by calculating the difference between the OS and the PFS curve at each time point. The time horizon in our model was 5 years in which 99.5% of patients would be dead in both treatment arms. The cycle length was set at 4 weeks as gemcitabine used 4 weeks as a cycle. This study was conducted from the Chinese healthcare system and US healthcare payer perspective. Life-years, quality-adjusted life-years (QALYs), overall costs, and incremental cost-effectiveness ratios (ICERs) between the treatments were measured. A 5% discount rate per year was applied for both cost and effectiveness in China (14) and a 3% in the US (15). The willingness-to-pay (WTP) threshold of Chinese Renminbi ¥72447 to ¥217341 per QALY gained (3× gross domestic product (GDP) per capita) was used in China (16). And for the US, the WTP threshold was US dollars $100000 to $150 000 per QALY gained (17).

Markov model is also widely used in the economic evaluation of drugs. However, there is no consensus regarding whether the Markov model is better or not when compared with the PS model. Both the Markov and PS models are recommended to assess model structure uncertainty (18). Therefore, we also developed a Markov model to validate our results. The Markov model was built with TreeAge Pro 2020. This model contained three mutually exclusive health states, including PFD, PD, and death. All patients entered the model from the PFD. From this state, they could then either stayed in the PFD, progress to the PD, or die. Patients in the PD could either remain in the same state or transition to death. Patients were assumed to receive SG or single-agent chemotherapy when they were in the PFD and discontinue when they transitioned to the PD or death. Time-dependent transition probabilities were used in this three-state Markov model. Calculation of transition probabilities based on the fitted PFS and OS models has been described in detail by Rui et al. (19). Transition probabilities from the PFD to death were assumed to be the same as the natural death rate, and the age-specific and sex-specific death rates were obtained from the life tables for China (20) and the US (21). The Markov model used the same cycle length, time horizon, costs, and utility values as the partitioned survival model.

### Clinical Data Inputs

**Figure 1:**
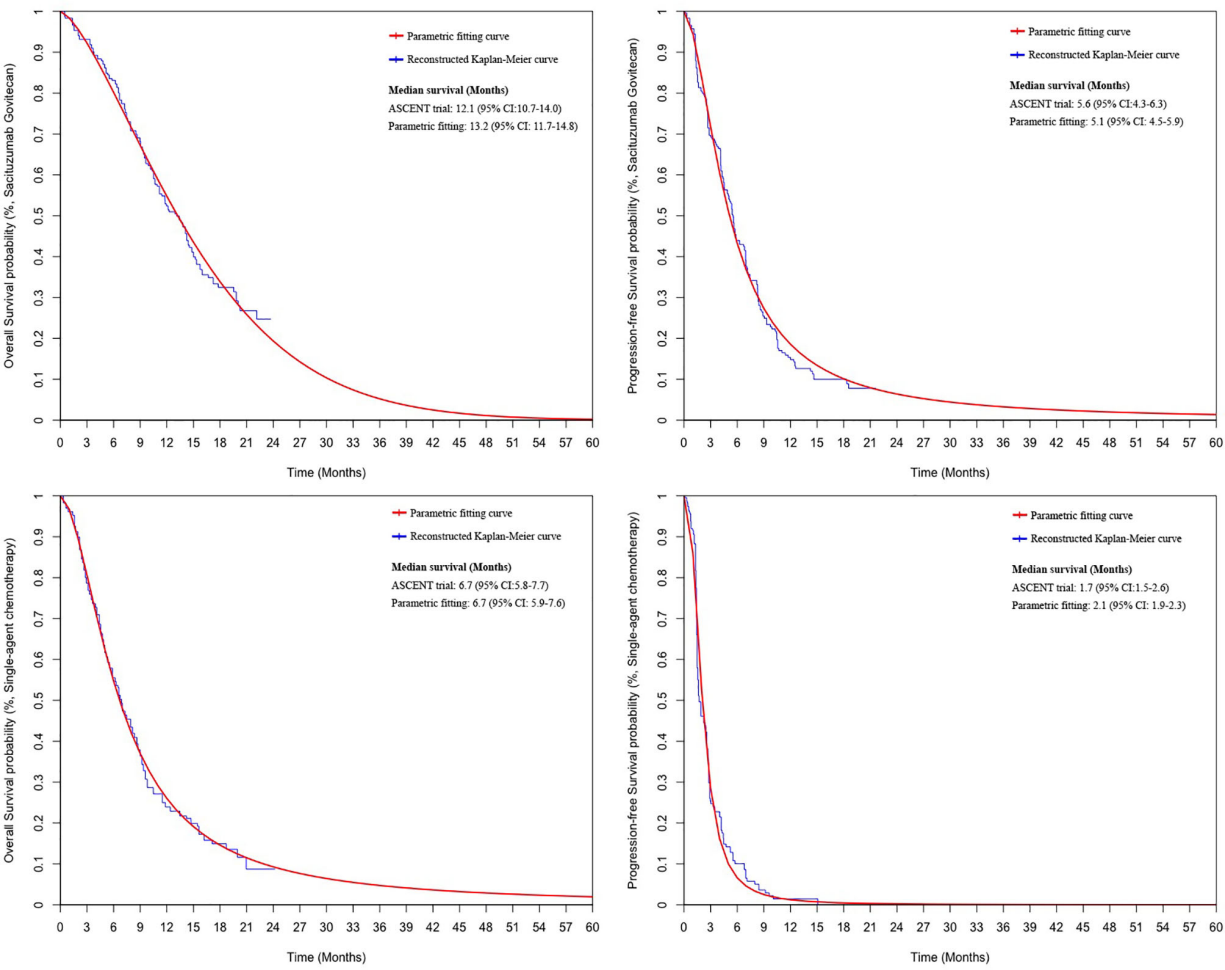
The comparison between the reconstructed Kaplan-Meier curves from the ASCENT trial and the best parametric fitting curves. CI: confidence interval.

PFS and OS curves for patients with SG or chemotherapy were modeled based on the results of the ASCENT trial (10) according to the standard statistical analyses described by Guyot et al. (22) and Baio et al. (23). The GetData Graph Digitizer (Version 2.26; http://getdata-graph-digitizer.com/) was used to gather the data points from the PFS and OS curves. These data points were then used to fit the following survival functions including gompertz, exponential, gamma, genf, gengamma, weibull, weibullPH, loglogistic, and lognormal. The function with the best fit was determined by the Akaike information criterion, Bayesian information criterion (24), and graphical validation (**Supplemental Figures 1-4**). We determined that the weibull and loglogistic model were the most reasonable functions for extrapolating OS and PFS in the SG arm and loglogistic model was the best for that in the chemotherapy arm (**Supplemental Table 2**). The parameters of fitting models are provided in **Table 1**. The comparison between the reconstructed Kaplan-Meier curves from the ASCENT trial and parametric fitting curves is presented in **Figure 1**.

**Table 1:** Basic parameters input to the model and the ranges for sensitivity analyses.

Parameters	Expected value	Range	Distribution	Source
**Clinical Data**				
Weibull OS survival model of SG	Shape: 1.447; Scale: 17.034	–	–	Model fitting
Loglogistic OS survival model of chemotherapy	Shape: 1.783; Scale: 6.675	–	–	Model fitting
Loglogistic PFS survival model of SG	Shape: 1.741; Scale: 5.133	–	–	Model fitting
Loglogistic PFS survival model of chemotherapy	Shape: 2.499; Scale: 2.076	–	–	Model fitting
**Cost in China (Chinese Renminbi ¥)**				
SG	192.5 per mg	114.4-240.6	Gamma	DrugsHK (25)
Eribulin	5277 per mg	3958-6596	Gamma	Tuling (26)
Vinorelbine	13.5 per 10 mg	10.1-16.9	Gamma	Tuling (26)
Capecitabine	2.8 per 500 mg	2.1-3.5	Gamma	SMPA (27)
Gemcitabine	158.5 per 1000 mg	118-198	Gamma	SMPA (27)
Drug administration	148 per month	111-185	Gamma	Huang et al. (28)
Follow-up	1041 per time	781-1302	Gamma	Zhang et al. (29); Liao et al. (30); Weng et al. (31)
Management of severe AE				
Neutropenia	2877 per event	2158-3597	Gamma	Ding et al. (32)
Anemia	6298 per event	4723-7872	Gamma	Dranitsaris et al. (33)
Leukopenia	2877 per event	2158-3597	Gamma	Ding et al. (32)
Thrombocytopenia	1069 per event	802-1336	Gamma	Dranitsaris et al. (33)
Diarrhea	4152 per event	3114-5190	Gamma	Dranitsaris et al. (33)
Nausea/Vomiting	323 per event	208-398	Gamma	Rui et al. (19); Hurley et al. (34)
Febrile neutropenia	4283 per event	3213-5354	Gamma	Dranitsaris et al., (33)
Best supportive care	10325 per event	7465-14755	Gamma	Rui et al. (19); Hurley et al. (34)
End-of-life care	15879 per event	6166-42411	Gamma	Rui et al. (19); Hurley et al. (34)
**Cost in the US (US dollar $)**				
SG	11.2 per mg	8.4-14	Gamma	CMS (35)
Eribulin	1177 per 1 mg	883-1471	Gamma	CMS (35)
Vinorelbine	9.5 per 10 mg	7.1-11.9	Gamma	CMS (35)
Capecitabine	2.4 per 500 mg	1.8-3	Gamma	CMS (35)
Gemcitabine	19.8 per 1000 mg	14.9-24.8	Gamma	CMS (35)
Drug administration	683 per month	512-853	Gamma	Kruse et al. (36)
Follow-up	1319 per time	989-1648	Gamma	Sorensen et al. (37)
Management of severe AE				
Neutropenia	9497 per event	7123-11871	Gamma	Rashid et al. (38)
Anemia	13110 per event	9832-16387	Gamma	Rashid et al. (38)
Leukopenia	9497 per event	7123-11871	Gamma	Rashid et al. (38)
Thrombocytopenia	11546 per event	8660-14433	Gamma	Sorensen et al. (37)
Diarrhea	3866 per event	2899-4832	Gamma	Sorensen et al. (37)
Nausea/Vomiting	3876 per event	2907-4346	Gamma	Sorensen et al. (37)
Febrile neutropenia	22814 per event	17110-28517	Gamma	Mistry et al. (39)
Best supportive care	4797	3598-5996	Gamma	Mistry et al. (39)
End-of-life care	9584	7188-11980	Gamma	Zhang et al. (40)
Probability of AE among patients with SG treatment				
Neutropenia	0.512	0.451-0.573	Beta	Bardia et al. (10)
Anemia	0.078	0.045-0.110	Beta	Bardia et al. (10)
Leukopenia	0.101	0.064-0.138	Beta	Bardia et al. (10)
Thrombocytopenia	0.016	0-0.031	Beta	Bardia et al. (10)
Diarrhea	0.105	0.067-0.142	Beta	Bardia et al. (10)
Nausea/Vomiting	0.039	0.015-0.062	Beta	Bardia et al. (10)
Febrile neutropenia	0.058	0.03-0.087	Beta	Bardia et al. (10)
**Probability of AE among patients with chemotherapy**				
Neutropenia	0.33	0.269-0.392	Beta	Bardia et al. (10)
Anemia	0.049	0.021-0.077	Beta	Bardia et al. (10)
Leukopenia	0.054	0.024-0.083	Beta	Bardia et al. (10)
Thrombocytopenia	0.013	0-0.028	Beta	Bardia et al. (10)
Diarrhea	0.004	0-0.013	Beta	Bardia et al. (10)
Nausea/Vomiting	0.004	0-0.013	Beta	Bardia et al. (10)
Febrile neutropenia	0.022	0.003-0.042	Beta	Bardia et al. (10)
Average weight of Chinses female	57.3 kg	43-71.6	Normal	NHC (41)
Average body surface of Chinese female*	1.57 m^2^	1.18-1.96	Normal	NHC (41)
Average weight of the US female	77.5 kg	58.1-96.9	Normal	CDC (42)
Average body surface of the US female*	1.86 m^2^	1.40-2.33	Normal	CDC (42)
**Utility**				
Progression-free disease	0.85	0.64-1	Beta	Wu et al. (43)
Progression of the disease	0.52	0.39-0.65	Beta	Wu et al. (43)
Disutility due to severe AE	0.28	0.21-0.35	Beta	Wu et al. (43)

AE, Adverse events; CDC, Centers for Disease Control; CMS, Centers for Medicare & Medicaid Services; NHC, National Health Commission of China; OS, Overall survival; PFS, Progression-free survival; SG, Sacituzumab Govitecan; US, United States. *The body surface was calculated based on the height and weight according to the Mosteller formula.

### Costs

The costs were estimated from the Chinese and US perspective and only direct medical costs were considered, including costs for drugs, drug administration, follow-up, severe adverse events (AEs) management, best supportive care, and end-of-life care. The average body weight or body surface area of the Chinese and US women were used to calculate the dosage of drugs because women accounted for more than 99% percent of all participants in the ASCENT trial (10). Since SG is currently not available on the mainland Chinese market, we used the Hongkong price (DrugsHK, www.drugshk.com) to estimate the likely price as many mainlanders would go to Hongkong for drugs due to the lower prices and easy accessibility. The price of SG in the US is from the literature (11). According to the ASCENT trial, 54% of patients used eribulin, 20% used vinorelbine, 13% used capecitabine, and 12% used gemcitabine for the single-agent chemotherapy. We used these proportions to estimate the costs of chemotherapy. The price of chemotherapy in China was based on the median price from the widely used Chinese Drug Price Database (Tuling, www.315jiage.cn). Moreover, capecitabine and gemcitabine have entered the centralized drugs procurement program list in China and their mean prices were obtained from the official medical procurement network (Sunshine Medical Procurement All-in-one, http://www.smpaa.cn/). Chemotherapy costs in the US were obtained from the Centers for Medicare & Medicaid Services (35). Costs of drug administration, follow-up, severe AEs management, best supportive care, and end-of-life care were derived from previously published studies (**Table 1**). The composition of follow-up costs included outpatient visit, laboratory evaluation, and imaging examination (29–31). According to the ASCENT trial, the computed tomography or magnetic resonance imaging examination was performed every 6 weeks for 36 weeks, then every 9 weeks thereafter, until disease progression leading to treatment discontinuation (10). We assigned follow-up costs to different cycles according to this follow-up pattern. Patients were assumed to have continuous best supportive care when diseases progressed and they also received end-of-life care before death. All costs were converted to 2020 values according to the local Consumer Price Index (44, 45).

### Utilities

Utility values of different health states were not reported in the ASCENT trial. We obtained the utility values from a recent similar cost-effectiveness analysis conducted by Wu et al. in which PFD, PD, and death state were assigned a value of 0.85, 0.578, and 0, respectively (43). According to Wu et al., the utility values in non-TNBC and TNBC were comparable as the quality of life was mainly affected by cancer stage but not the hormone status (43, 46). Then they estimated the utility values for PFD and PD based on values in non-TNBC (40, 47). Patients with severe AEs were assumed to have a disutility of 0.28 and all these AEs were assumed to have been incurred in the first cycle (43).

### Sensitivity and Subgroup Analyses

To assess the robustness of the model, one-way sensitivity analyses were performed on all parameters. The range of each parameter used in the one-way sensitivity analyses was based on either the reported 95% confidence interval (CI) in the referenced literature or a ± 25% change from the base case value. Probabilistic sensitivity analysis with a Monte Carlo simulation (10 000 iterations) was performed by simultaneously sampling the key model parameters from the prespecified distributions. All the costs were assigned with a gamma distribution and probability, proportion, and utilities were assigned with a beta distribution. A cost-effectiveness acceptability curve based on the results from 10000 iterations was created to evaluate the likelihood the SG would be considered cost-effective at different WTP thresholds.

We also performed the subgroup analyses to investigate the uncertainty of economic outcomes caused by the subgroups reported in the ASCENT trial. The ICER was calculated for each subgroup by using the reported subgroup-specific HRs for OS and PFS. The input data were assumed to be the same for all subgroups except for the HRs for OS and PFS. Details about subgroup analyses were described in Supplemental Material.

## Results

### Base Case Results

**Table 2** shows the results of the base case analysis. From the Chinese perspective, the PS model predicted that SG yielded an additional 0.35 QALY at an additional cost of ¥2257842. The ICER was ¥6375856 ($924037)/QALY. From the US perspective, SG yielded the same additional QALY at an extra cost of $175393 and the ICER was $494479/QALY. For the Markov model, the ICER was ¥6407626 ($928641)/QALY in China and $507416/QALY in the US. Results from the PS model and Markov model were quite similar.

**Table 2:** Base case results with PS model and Markov model from the Chinese and US perspectives.

Factor	Chinese perspective (PS model)			US perspective (PS model)		
	SG	Chemotherapy	Differences	SG	Chemotherapy	Differences
LYs	1.28	0.87	0.41	1.28	0.87	0.41
QALYs	0.87	0.52	0.35	0.87	0.52	0.35
Drug costs	¥2305982	¥439794	¥2266188	$181706	$10633	$171073
Overall Costs	¥2501955	¥244112	¥2257842	$304393	$129000	$175393
ICER			¥6375856 /QALY			$501123 /QALY
	**Chinese perspective (Markov model)**			**US perspective (Markov model)**		
**Factor**	**SG**	**Chemotherapy**	**Differences**	**SG**	**Chemotherapy**	**Differences**
LYs	1.24	0.84	0.40	1.27	0.89	0.38
QALYs	0.85	0.50	0.35	0.86	0.53	0.33
Drug costs	¥2261619	¥39319	¥2222300	$181162	$10556	$170606
Overall Costs	¥2449455	¥235355	¥2214100	$300940	$131205	$169735
ICER			¥6407626 /QALY			$507416 /QALY

ICER, Incremental cost-effectiveness ratio; LYs, Life-years; PS, Partitioned survival model; QALYs, Quality-adjusted life-years; SG, Sacituzumab Govitecan; US, United States.

In China, when SG costs 30%, 20%, and 10% of its current price, the ICERs were ¥1817599 ($263420)/QALY, ¥1166419 ($169046)/QALY, and ¥515239 ($74672)/QALY, respectively. In the US, when SG costs 30%, 20%, and 10% of the current price, the ICERs were $135887/QALY, $84660/QALY, and $33432/QALY, respectively.

### One-Way Sensitivity Analysis

**Figure 2:**
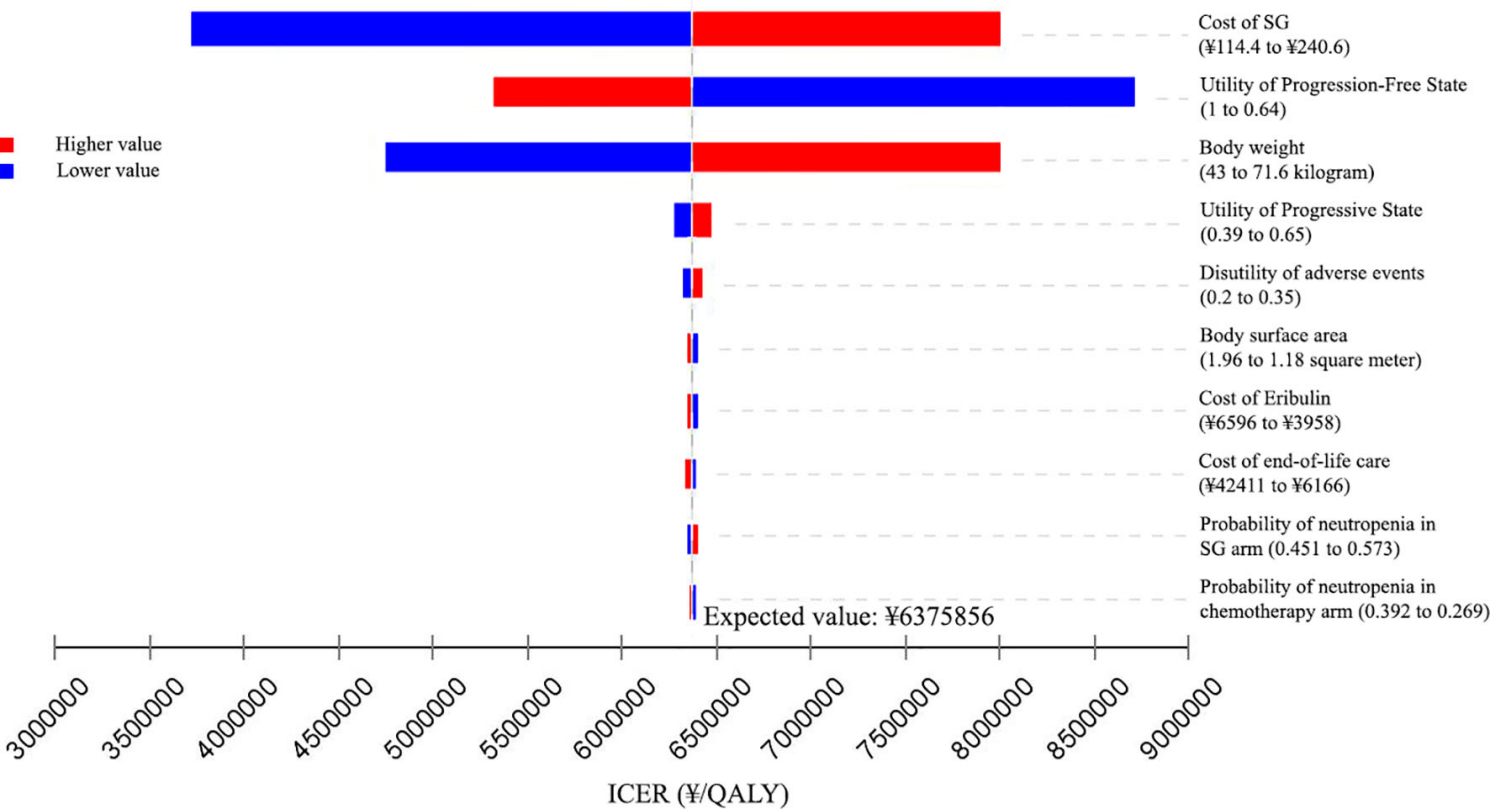
Tornado diagram of one-way sensitivity analyses of Sacituzumab Govitecan *versus* single-agent chemotherapy in the treatment of metastatic triple-negative breast cancer from the Chinese perspective. ICER, incremental cost-effectiveness ratio; QALY, quality-adjusted life-years; SG, Sacituzumab Govitecan.

The results of the one-way sensitivity analyses are presented in Tornado diagrams, showing the effect from uncertainty in parameter input on the base case of ICER. The results were sensitive to the cost of SG, the utility of PFD, and body weight. From the Chinese perspective (**Figure 2**), when the cost of SG was reduced to 75% of the baseline, the ICER fell to ¥4747907 ($688102)/QALY. When the cost of SG was increased to 125% of the baseline, the ICER was ¥8003805 ($1159972)/QALY. Across the broad variation in the ranges for all input parameters, the ICERs were greater than the Chinese WTP threshold of ¥217341/QALY.

**Figure 3:**
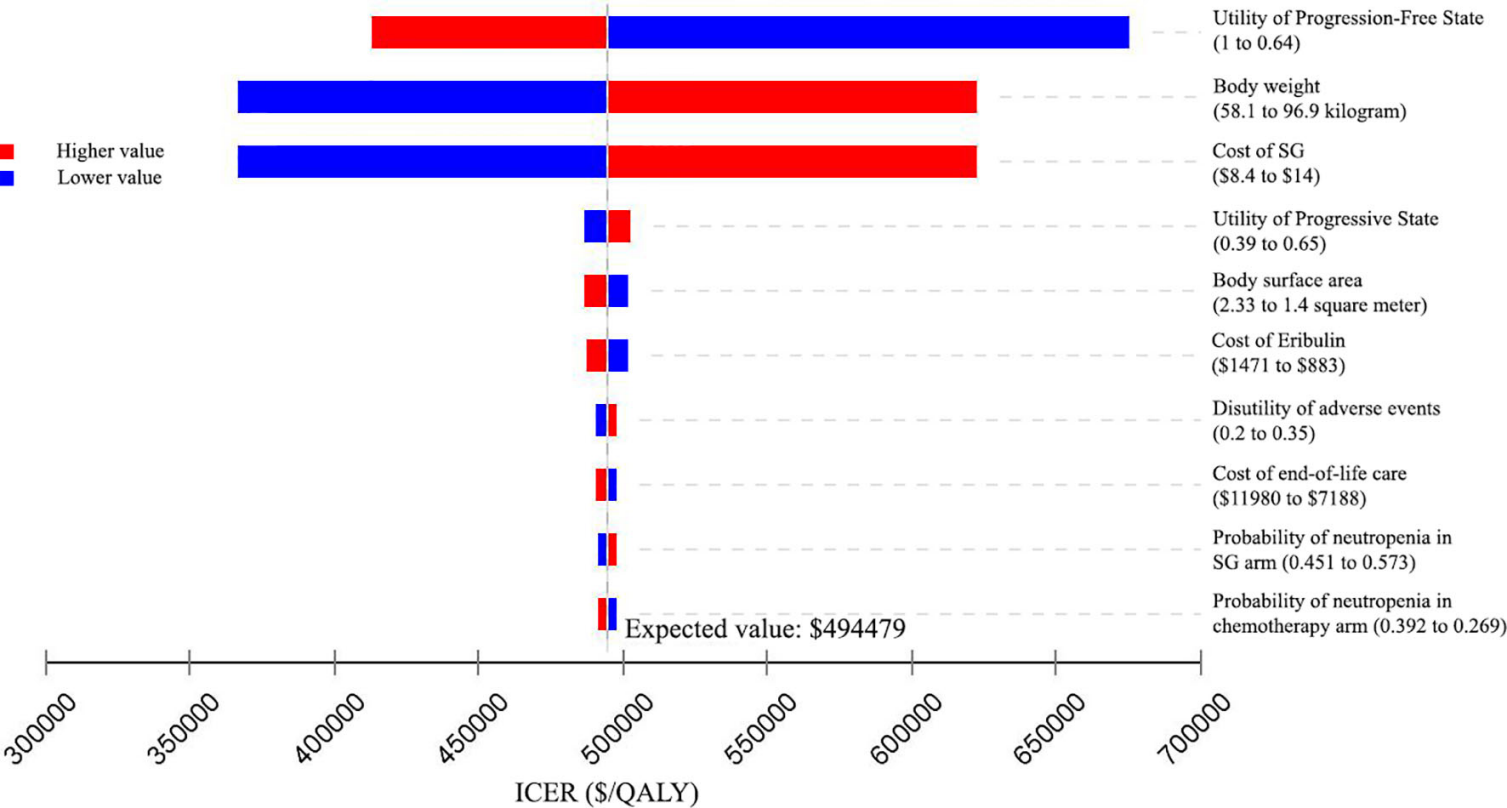
Tornado diagram of one-way sensitivity analyses of Sacituzumab Govitecan *versus* single-agent chemotherapy in the treatment of metastatic triple-negative breast cancer from the United States perspective. ICER: incremental cost-effectiveness ratio; QALY: quality-adjusted life-year; SG: Sacituzumab Govitecan.

Similar results were obtained from the US perspective (**Figure 3**), When the cost of SG was reduced to 75% of the baseline, the ICER fell to $366410/QALY. When the cost of SG was increased to 125% of the baseline, the ICER was $622548/QALY. The ICERs were all greater than the US WTP threshold of ¥150000/QALY when the input parameters varied in their ranges.

### Probabilistic Sensitivity Analysis

**Figure 4:**
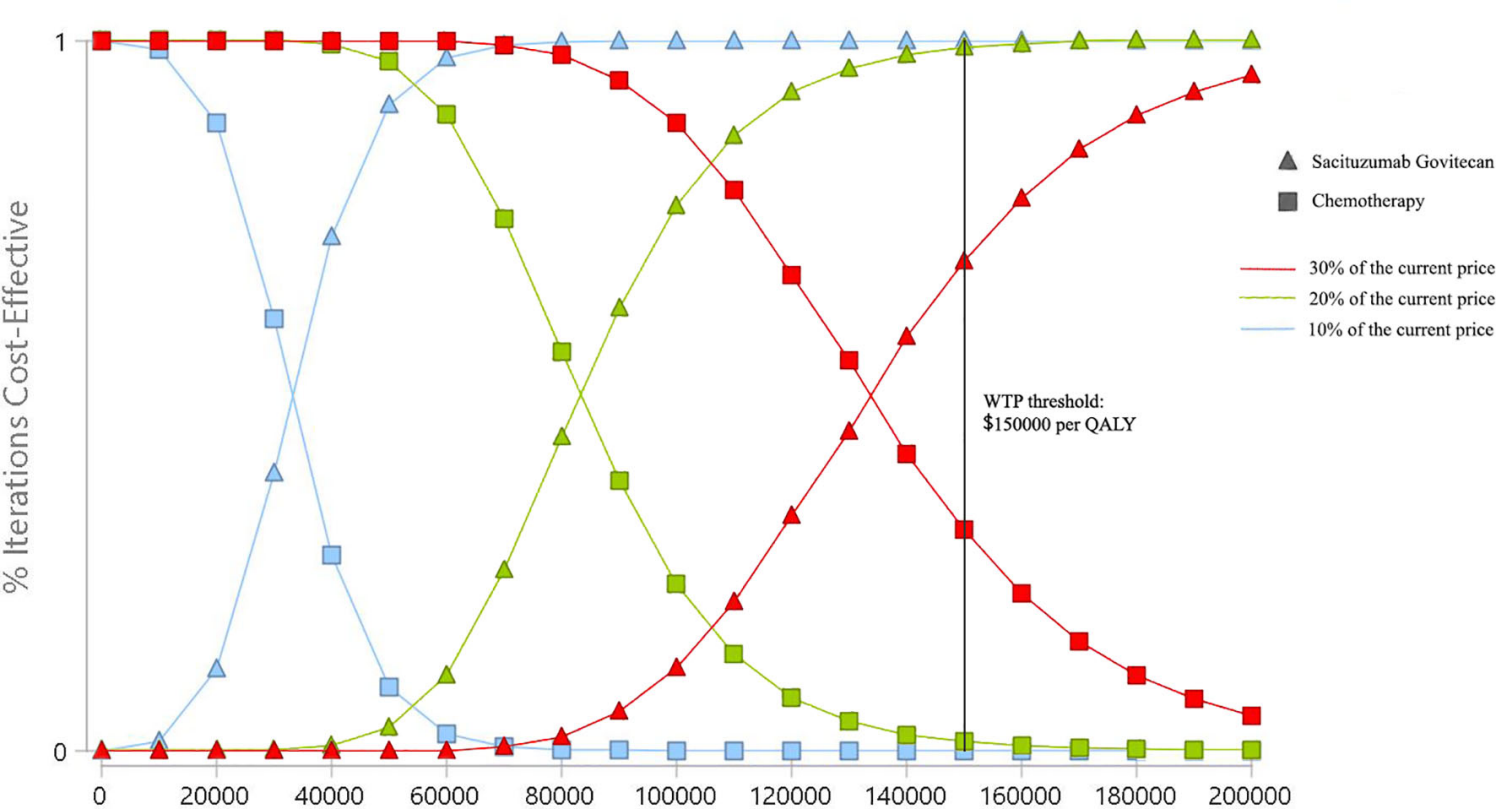
Cost-effectiveness acceptability curves of Sacituzumab Govitecan *versus* single-agent chemotherapy in the treatment of metastatic triple-negative breast cancer from the United States payer perspective. QALY, quality-adjusted life-year; WTP, willingness-to-pay.

At the WTP threshold of ¥217341/QALY in China, the cost-effective probability of SG treatment was 0%. When the unit price of SG was 30%, 20%, and 10% of the current price, the probabilities of cost-effectiveness for SG treatment were still 0%. When the cost was reduced to ¥10.44/mg (5.4% of the current price), the probability of cost-effectiveness of SG treatment increased to 50%. From the US perspective, with the same strategy of price reduction, the cost-effective probabilities of SG treatment were 69.3%, 98.4%, and 100% (**Figure 4**). When the cost was reduced to $3.65/mg (32.6% of the current price), the probability of cost-effectiveness of SG treatment would increase to 50%.

### Subgroup Analysis

ICER range of different subgroups obtained by varying the HRs for OS and PFS was presented in **Supplemental Table 3**. In China, the lowest ICER among different subgroups was ¥3478680 ($504157)/QALY, which was above the WTP threshold of ¥217341/QALY. In the US, the lowest ICER among different subgroups was $366967/QALY and it was above the WTP threshold of ¥150000/QALY.

## Discussion

The huge demand for treating TNBC and the unmet need for a precise economic evaluation of SG is the motivation of the current study (11). Due to the different national conditions and medical environments, we performed an economic evaluation on the basis of the Chinese and US perspective. Our findings could provide useful economic information regarding the SG treatment. Moreover, SG is not available on the mainland Chinese market, and our study can provide a reference for the listing of SG in China in the future. According to the results, the ICER at base case estimate for SG *vs.* chemotherapy was ¥6375856 ($924037)/QALY in China and $494479/QALY in the US, which were significantly higher than the WTP threshold of ¥217341/QALY and $150000/QALY, respectively. Both sensitivity and subgroup analyses demonstrated that the model was robust. These findings indicate that SG is unlikely to be a cost-effective treatment for mTNBC both in China and the US.

One-way sensitivity analyses demonstrated that the price of SG (aside from the utility of progression-free state and average body weight) was the most influential factor in our study. Probabilistic sensitivity analyses showed that in China, SG had a 50% chance to be cost-effective when its cost was reduced to ¥10.44/mg (5.4% of the current price). In the US, when the cost of SG was $3.65/mg (32.6% of the current price), there was half the probability of cost-effectiveness of SG treatment would approximate to 50%. These results suggested that reducing the price of SG was essential to enhance the feasibility of using this regimen as a preferred treatment. Action has been taken by the government of two sides to reduce anti-cancer drug prices. For example, as part of its plan to reform the healthcare system, the Chinese government has launched the centralized drugs procurement program. The prices of many drugs dropped dramatically after they entered the procurement list (48). In the US, the government released American Patients First, its blueprint for cutting drug prices and reducing out-of-pocket payments (49). Significant price reduction or financial support is critical for patients to access innovative treatments.

The strengths of this study are worth highlighting. First, to our knowledge, this study is the first modeling analysis to evaluate the economic outcomes of SG treatment of mTNBC by incorporating the latest evidence. SG has received approval from the FDA while data on its economic outcomes are scarce. Moreover, even though SG has not been approved in China, the National Medical Products Administration has accepted for review the Biologics License Application for it in breast cancer (50). Clinical trial targeting Chinses patients is ongoing (ClinicalTrials.gov Identifier: NCT04454437). Second, a Markov model was constructed to validate the results of PS model and the results from both versions of the model were very close, proving the reliability of our results. Third, we conducted the subgroup analysis according to the different subgroups prespecified by the ASCENT trial (10). Economic information based on subgroups might be helpful in treatment decisions.

The limitations of this study should be noted. First, the clinical data were obtained from the ASCENT trial and the Asian population accounted for only 3.8% of the entire participants (10). This might not reflect the treatment effect of Chinese populations. Clinical trial targeting Chinese population is ongoing and the results are needed to validate our conclusion. Second, we did not have access to individual patient’s data and the health benefits were assumed through the fitting of parametric distributions to the reported Kaplan-Meier PFS and OS data, which might result in uncertainty in the model outputs. Moreover, in the subgroup analysis, the OS and PFS Kaplan-Meier curves in the specific subgroups were assumed to be the same as those in the overall population. Third, since SG has not been marketed in mainland China, the model’s drug price from the Chinese perspective is according to the Hongkong price. We calculated the 30, 20, and 10% off the model price to estimate the cost-effectiveness of SG. Fourth, since the quality of life or utility data weren’t reported by the ASCENT trial, we assumed they were similar to the previous study. We also assumed that the Chinese population had the same utilities as the West. However, a range of ± 25% of utility values was used in the sensitivity analyses to investigate the effect of changes on the results.

In conclusion, SG is unlikely to be a cost-effective treatment of mTNBC at the current price both in China and the US. Lowering the price of SG, generic drugs, and new payment systems are needed to improve its cost-effectiveness and our analysis provides valuable recommendations.

## Data Availability Statement

The original contributions presented in the study are included in the article/**Supplementary Material**. Further inquiries can be directed to the corresponding authors.

## Author Contributions

JC and MH were involved in the literature research, data collection, and manuscript preparation. BS were involved in the study design, model development, and economic analysis. AL was responsible for revising the and reviewing the manuscript. All the authors made the decision to submit the manuscript for publication.

## Funding

This work was supported by the Beijing Science and Technology Planning Project (No. Z181100009618035).

## Conflict of Interest

The authors declare that the research was conducted in the absence of any commercial or financial relationships that could be construed as a potential conflict of interest.

## Publisher’s Note

All claims expressed in this article are solely those of the authors and do not necessarily represent those of their affiliated organizations, or those of the publisher, the editors and the reviewers. Any product that may be evaluated in this article, or claim that may be made by its manufacturer, is not guaranteed or endorsed by the publisher.
