# Supplementary material for: Economic Evaluation of Sacituzumab Govitecan for the Treatment of Metastatic Triple-Negative Breast Cancer in China and the US
Source: Front Oncol. 2021 Oct 28;11:734594. doi: 10.3389/fonc.2021.734594 (PMC8581633; doi:10.3389/fonc.2021.734594)
Supplement: Supplementary file 1 [file DataSheet_1.docx]

Subgroup analyses

We conducted subgroup analyses for each subgroup based on the subgroup-specific HRs (SG versus single-agent chemotherapy) for OS and PFS [1-3]. According to our results of model fitting, the OS Kaplan-Meier curve in the overall population with SG could be fitted by the Weibull model, which was determined by its shape parameter (γ__) and scale parameter (λ_SG_). The OS curve in the specific subgroup with SG was assumed to be the same as that in the overall population with SG treatment. Then the parametric survival model fitting OS curve in this specific subgroup with single-agent chemotherapy could be obtained as follows: (1) the shape parameter was the same between two different treatments (γ_placebo_ =γ_SG_); (2) the scale parameter of the placebo subgroup was multiplied by the scale parameter of the SG subgroup and the subgroup-specific HR, that was λ_placebo_ = λ_SG*_HR. The same rules could be applied to the PFS curve. The range of the ICER in this subgroup was then calculated by varying the HRs for both OS and PFS simultaneously.

| Supplemental Table 1. CHEERS Checklist | | | |
| --- | --- | --- | --- |
| **Section** | **Item No** | **Recommendation** | **Reported** |
| **Title and Abstract** | | | |
| Title | 1 | Identify the study as an economic evaluation or use more specific terms such as “cost-effectiveness analysis, and describe the interventions compared. | Yes |
| Abstract | 2 | Provide a structured summary of objectives, perspective, setting, methods (including study design and inputs), results (including base case and uncertainty analyses), and conclusions. | Yes |
| **Introduction** | | | |
| Background and objectives | 3 | Provide an explicit statement of the broader context for the study.  Present the study question and its relevance for health policy or practice decisions. | Yes |
| **Methods** | | | |
| Target population and subgroups | 4 | Describe characteristics of the base case population and subgroups analyzed, including why they were chosen. | Yes |
| Setting and location | 5 | State relevant aspects of the system(s) in which the decision(s) need(s) to be made. | Yes |
| Study perspective | 6 | Describe the perspective of the study and relate this to the costs being evaluated. | Yes |
| Comparators | 7 | Describe the interventions or strategies being compared and state why they were chosen. | Yes |
| Time horizon | 8 | State the time horizon(s) over which costs and consequences are being evaluated and say why appropriate. | Yes |
| Discount rate | 9 | Report the choice of discount rate(s) used for costs and outcomes and say why appropriate. | Yes |
| Choice of health outcomes | 10 | Describe what outcomes were used as the measure(s) of benefit in the evaluation and their relevance for the type of analysis performed. | Yes |
| Measurement of effectiveness | 11a | Single study-based estimates: Describe fully the design features of the single effectiveness study and why the single study was a sufficient source of clinical effectiveness data. | Yes |
|  | 11b | Synthesis-based estimates: Describe fully the methods used for identification of included studies and synthesis of clinical effectiveness data. | NA |
| Measurement and valuation of preference based outcomes | 12 | If applicable, describe the population and methods used to elicit preferences for outcomes. | Yes |
| Estimating resources and costs | 13a | Single study-based economic evaluation: Describe approaches used to estimate resource use associated with the alternative interventions. Describe primary or secondary research methods for valuing each resource item in terms of its unit cost. Describe any adjustments made to approximate to opportunity costs. | NA |
|  | 13b | Model-based economic evaluation: Describe approaches and data sources used to estimate resource use associated with model health states. Describe primary or secondary research methods for valuing each resource item in terms of its unit cost. Describe any adjustments made to approximate to opportunity costs. | Yes |
| Currency, price date, and conversion | 14 | Report the dates of the estimated resource quantities and unit costs. Describe methods for adjusting estimated unit costs to the year of reported costs if necessary. Describe methods for converting costs into a common currency base and the exchange rate | Yes |
| Choice of model | 15 | Describe and give reasons for the specific type of decision-analytical model used. Providing a figure to show model structure is strongly recommended. |  |
| Assumptions | 16 | Describe all structural or other assumptions underpinning the decision-analytical model. | Yes |
| Analytical methods | 17 | Describe all analytical methods supporting the evaluation. This could include methods for dealing with skewed, missing, or censored data; extrapolation methods; methods for pooling data; approaches to validate or make adjustments (such as half cycle corrections) to a model; and methods for handling population heterogeneity and uncertainty. | Yes |
| **Results** | | | |
| Study parameters | 18 | Report the values, ranges, references, and, if used, probability distributions for all parameters. Report reasons or sources for distributions used to represent uncertainty where appropriate. Providing a table to show the input values is strongly recommended. | Yes |
| Incremental costs and outcomes | 19 | For each intervention, report mean values for the main categories of estimated costs and outcomes of interest, as well as mean differences between the comparator groups. If applicable, report incremental cost-effectiveness ratios. | Yes |
| Characterizing uncertainty | 20a | Single study-based economic evaluation: Describe the effects of sampling uncertainty for the estimated incremental cost and incremental effectiveness parameters, together with the impact of methodological assumptions (such as discount rate, study perspective). | NA |
|  | 20b | Model-based economic evaluation: Describe the effects on the results of uncertainty for all input parameters, and uncertainty related to the structure of the model and assumptions. | Yes |
| Characterizing heterogeneity | 21 | If applicable, report differences in costs, outcomes, or cost-effectiveness that can be explained by variations between subgroups of patients with different baseline characteristics or other observed variability in effects that are not reducible by more information. | Yes |
| **Discussion** | | | |
| Study findings, limitations, generalizability, and current knowledge | 22 | Summarize key study findings and describe how they support the conclusions reached. Discuss limitations and the generalisability of the findings and how the findings fit with current knowledge. | Yes |
| **Other** | | | |
| Source of funding | 23 | Describe how the study was funded and the role of the funder in the identification, design, conduct, and reporting of the analysis. Describe other non- monetary sources of support. | Yes |
| Conflicts of interest | 24 | Describe any potential for conflict of interest of study contributors in accordance with journal policy. In the absence of a journal policy, we recommend authors comply with International Committee of Medical Journal Editors recommendations. | Yes |

| Supplemental Table 2. AIC scores and BIC scores for parametric models | | | | | | | | |
| --- | --- | --- | --- | --- | --- | --- | --- | --- |
| Parametric models | OS of SG | | OS of chemotherapy | | PFS of SG | | PFS of chemotherapy | |
|  | AIC | BIC | AIC | BIC | AIC | BIC | AIC | BIC |
| Gompertz | 1140.537 | 1147.456 | 1166.826 | 1173.728 | 1151.515 | 1158.434 | 768.187 | 775.089 |
| Exponential | 1154.184 | 1157.643 | 1167.537 | 1170.988 | 1151.088 | 1154.548 | 771.874 | 775.326 |
| Gamma | 1133.229 | 1140.148 | 1152.608 | 1159.510 | 1139.096 | 1146.016 | 720.237 | 727.139 |
| Genf | 1133.346 | 1147.184 | 1150.408 | 1164.213 | 1138.448 | 1152.286 | - | - |
| Gengamma | 1134.958 | 1145.337 | 1150.340 | 1160.693 | 1136.888 | 1147.266 | 702.498 | 712.851 |
| Weibull | **1133.101** | **1140.020** | 1156.434 | 1163.336 | 1142.558 | 1149.477 | 738.065 | 744.967 |
| WeibullPH | 1133.101 | 1140.020 | 1156.434 | 1163.336 | 1142.558 | 1149.477 | 738.065 | 744.967 |
| Loglogistic | 1134.439 | 1141.359 | **1146.660** | **1153.562** | **1136.476** | **1143.395** | **693.518** | **700.420** |
| Lognormal | 1146.190 | 1153.109 | 1151.946 | 1158.848 | 1139.678 | 1146.597 | 700.519 | 707.421 |
| AIC: Akaike information criterion; BIC: Bayesian information criterion; OS: Overall survival; PFS: Progression-free survival; SG: Sacituzumab Govitecan | | | | | | | | |

| Supplemental Table 3. Summary of subgroup analyses | | | | |
| --- | --- | --- | --- | --- |
| The Chinese perspective | | | | |
| Subgroup | HR for OS  (95% CI) | HR for PFS  (95% CI) | ICER,  ¥/QALY | Cost-effectiveness probability of SG, % |
| Age, years |  |  |  |  |
| < 65 | 0.50 (0.40-0.64) | 0.46 (0.35-0.59) | 4463251-7187475 | 0 |
| ≥ 65 | 0.37 (0.22-0.64) | 0.22 (0.12-0.40) | 3478680-6384678 | 0 |
| Prior Therapies |  |  |  |  |
| 2 or 3 | 0.44 (0.34-0.56) | 0.39 (0.29-0.52) | 4094103-5978133 | 0 |
| >3 | 0.60 (0.40-0.89) | 0.48 (0.32-0.72) | 4412320-18222308 | 0 |
| Prior PD-1 inhibitors use |  |  |  |  |
| Yes | 0.52 (0.34-0.77) | 0.37 (0.24-0.57) | 4023681-9596224 | 0 |
| No | 0.47 (0.37-0.61) | 0.42 (0.32-0.56) | 4269713-6662492 | 0 |
| Liver Metastasis |  |  |  |  |
| Yes | 0.51 (0.37-0.70) | 0.48 (0.34-0.67) | 4300612-8683910 | 0 |
| No | 0.45 (0.33-0.60) | 0.36 (0.26-0.50) | 4011777-6322417 | 0 |
| Initial diagnosis of TNBC |  |  |  | 0 |
| Yes | 0.50 (0.38-0.65) | 0.38 (0.29-0.51) | 4268428-6966160 | 0 |
| No | 0.44 (0.30-0.64) | 0.48 (0.32-0.72) | 3970812-7804198 | 0 |
| The US perspective | | | | |
| Subgroup | HR for OS  (95% CI) | HR for PFS  (95% CI) | ICER,  $/QALY | Cost-effectiveness probability of SG, % |
| Age, years |  |  |  |  |
| < 65 | 0.50 (0.40-0.64) | 0.46 (0.35-0.59) | 449084-631720 | 0 |
| ≥ 65 | 0.37 (0.22-0.64) | 0.22 (0.12-0.40) | 379206-531743 | 0 |
| Race |  |  |  |  |
| White | 0.45 (0.36-0.58) | 0.39 (0.30-0.51) | 429887-557194 | 0 |
| Black | 0.64 (0.34-1.21) | 0.45 (0.24-0.86) | 413267-negative | 0 |
| Asian | 0.38 (0.11-1.28) | 0.40 (0.08-2.08) | 366967-negative | 0 |
| Prior Therapies |  |  |  |  |
| 2 or 3 | 0.44 (0.34-0.56) | 0.39 (0.29-0.52) | 424721-552501 | 0 |
| >3 | 0.60 (0.40-0.89) | 0.48 (0.32-0.72) | 441140-1291384 | 0 |
| Prior PD-1 inhibitor use |  |  |  |  |
| Yes | 0.52 (0.34-0.77) | 0.37 (0.24-0.57) | 413267-738887 | 0 |
| No | 0.47 (0.37-0.61) | 0.42 (0.32-0.56) | 436278-596384 | 0 |
| Liver Metastasis |  |  |  |  |
| Yes | 0.51 (0.37-0.70) | 0.48 (0.34-0.67) | 441208-738878 | 0 |
| No | 0.45 (0.33-0.60) | 0.36 (0.26-0.50) | 416634-561988 | 0 |
| Initial diagnosis of TNBC |  |  |  |  |
| Yes | 0.50 (0.38-0.65) | 0.38 (0.29-0.51) | 430323-593538 | 0 |
| No | 0.44 (0.30-0.64) | 0.48 (0.32-0.72) | 425545-708715 | 0 |
| CI: Confidence interval; HR: Hazard ratio; ICER: Incremental cost-effectiveness ratio; OS: Overall survival; PD: Programmed death; PFS: Progression-free survival; QALY: Quality-adjusted life-years; TNBC: Triple negative breast cancer  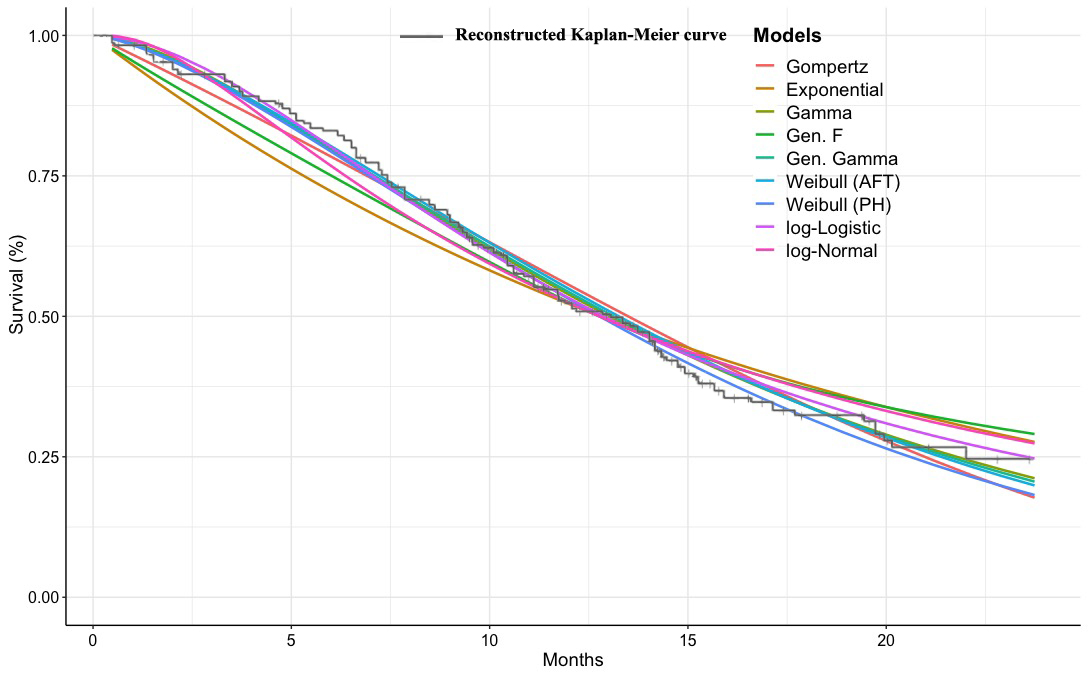 | | | | |

Figure S1. Comparison between reconstructed Kaplan-Meier curve for overall survival and all the parametric fitting curves among patients with Sacituzumab Govitecan.


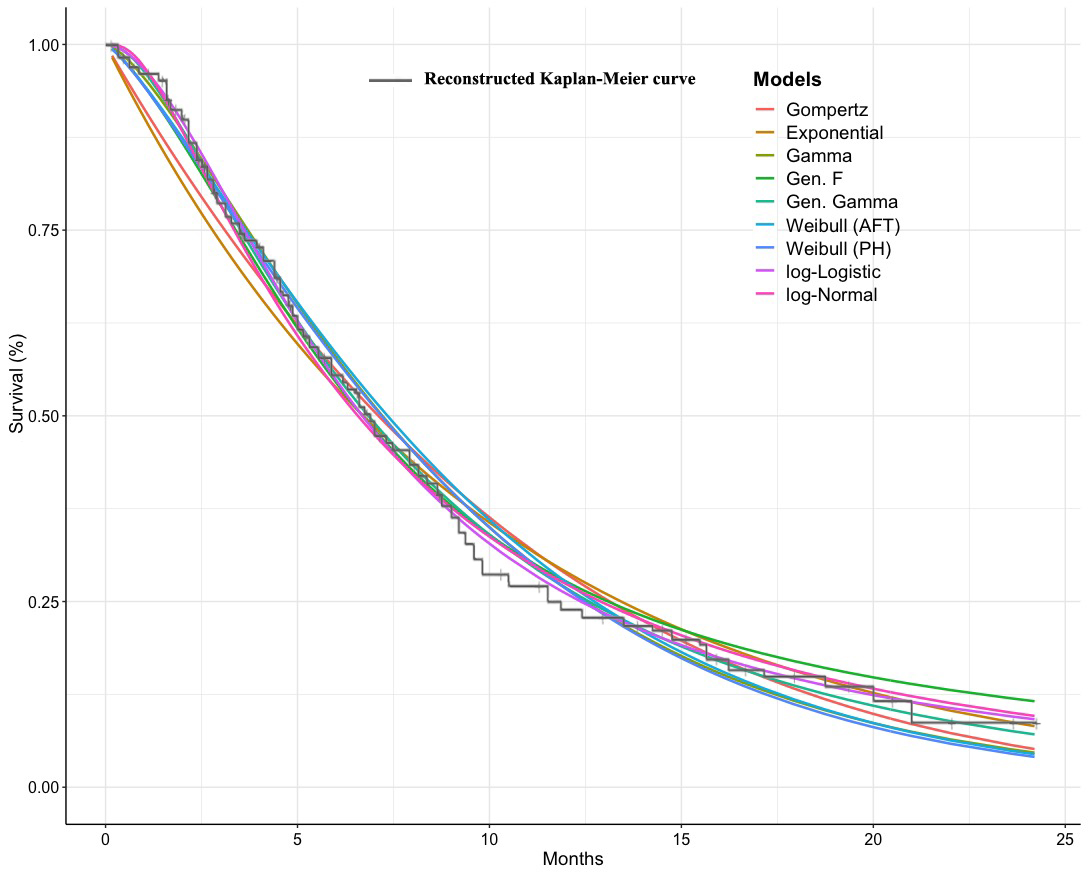


Figure S2. Comparison between reconstructed Kaplan-Meier curve for overall survival and all the parametric fitting curves among patients with single-agent chemotherapy.


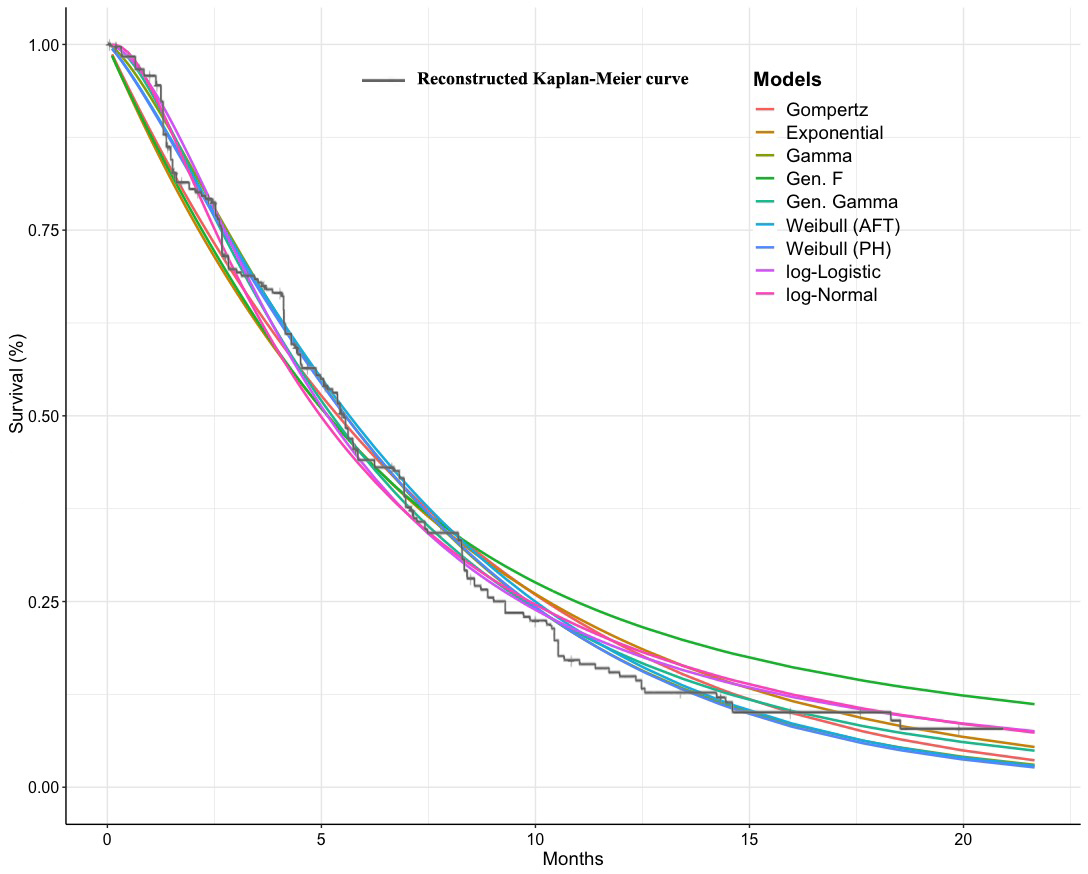


Figure S3. Comparison between reconstructed Kaplan-Meier curve for progression-free survival and all the parametric fitting curves among patients with Sacituzumab Govitecan.


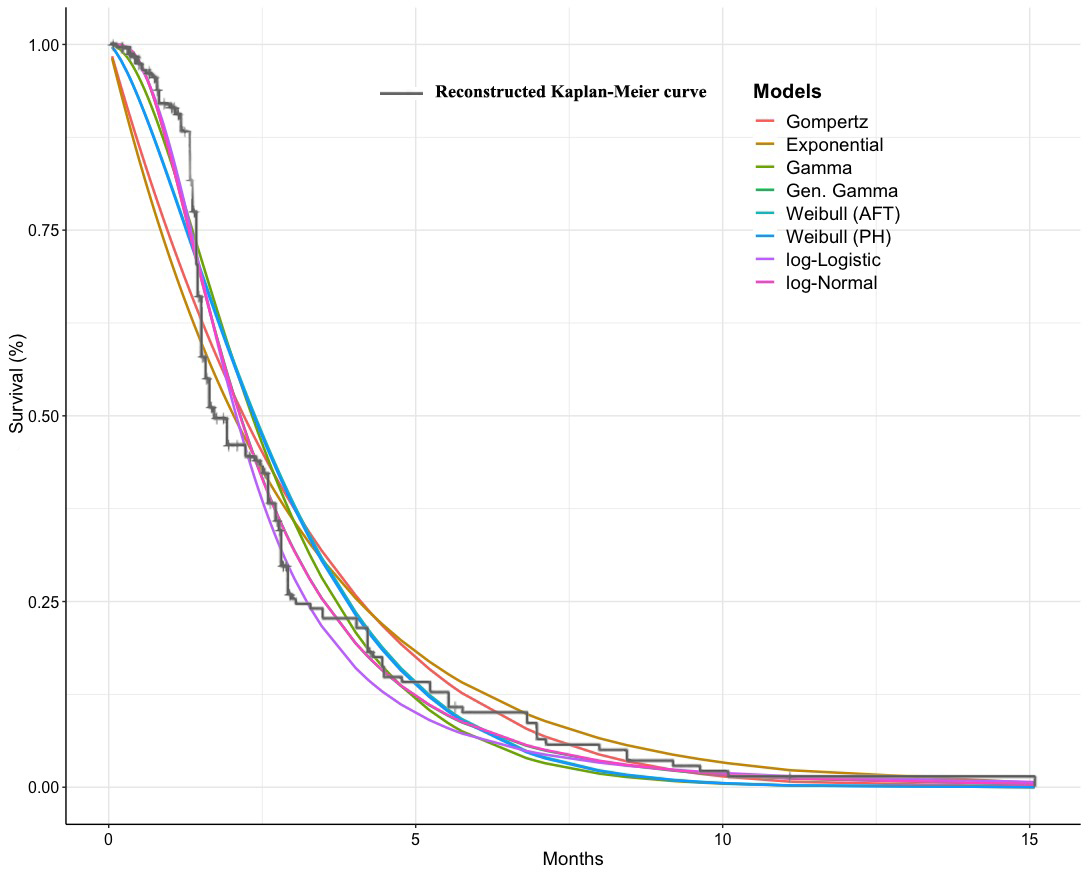


Figure S4. Comparison between reconstructed Kaplan-Meier curve for progression-free survival and all the parametric fitting curves among patients with single-agent chemotherapy.

Reference

1. Pei, R., et al., *Nivolumab vs Pembrolizumab for Treatment of US Patients With Platinum-Refractory Recurrent or Metastatic Head and Neck Squamous Cell Carcinoma: A Network Meta-analysis and Cost-effectiveness Analysis.* JAMA network open, 2021. **4**(5): p. e218065-e218065.

2. Wu, B. and F. Ma, *Cost-effectiveness of adding atezolizumab to first-line chemotherapy in patients with advanced triple-negative breast cancer.* Therapeutic Advances in Medical Oncology, 2020. **12**: p. 1758835920916000.

3. Su, D., B. Wu, and L. Shi, *Cost-effectiveness of Atezolizumab Plus Bevacizumab vs Sorafenib as First-Line Treatment of Unresectable Hepatocellular Carcinoma.* JAMA network open, 2021. **4**(2): p. e210037-e210037.
