# Supplementary material for: Economic Evaluation of Sacituzumab Govitecan for the Treatment of Metastatic Triple-Negative Breast Cancer in China and the US
Source: Front Oncol. 2021 Oct 28;11:734594. doi: 10.3389/fonc.2021.734594 (PMC8581633; doi:10.3389/fonc.2021.734594)
Supplement: Supplementary file 3 [file DataSheet_3.docx]

# required packages

library(survival)

library(survminer)

library(survHE)

#building IPD data for OS curve with SG treatment

surv_inp<-"SG_OS.txt"

nrisk_inp<-"nrisk_SG_OS.txt"

km_out<-"KM_SG_OS.txt"

ipd_out<-"IPD_SG_OS.txt"

digitise(surv_inp,nrisk_inp,km_output = "KM_SG_OS.txt",ipd_output = "IPD_SG_OS.txt")

SG_OS<-make.ipd(list("IPD_SG_OS.txt"),var.labs = c("time","event","arm"))

#building IPD data for OS curve with placebo treatment

surv_inp<-"placebo_OS.txt"

nrisk_inp<-"nrisk_placebo_OS.txt"

km_out<-"KM_placebo_OS.txt"

ipd_out<-"IPD_placebo_OS.txt"

digitise(surv_inp,nrisk_inp,km_output = "KM_placebo_OS.txt",ipd_output = "IPD_placebo_OS.txt")

placebo_OS<-make.ipd(list("IPD_placebo_OS.txt"),var.labs = c("time","event","arm"))

#plot reconstructed curves

OS_data<-make.ipd(list("IPD_SG_OS.txt", "IPD_placebo_OS.txt"),var.labs = c("time","event","arm"))

fit_OS<-survfit(Surv(time,event == 1)~arm,data = OS_data)

ggsurvplot(fit_PFS,data = PFS_data,

pval = TRUE,

conf.int = TRUE,

risk.table = TRUE,

palette = c("#D95F02","steelblue"),

legent.labs=c("placebo","SG"),

risk.table.height=0.2,

break.time.by = 3。

xlab=c("Months"),

ylab=c("Survival"),

risk.table.col="strata")

# fit parametric survival models with reconstructed curves

mods<-c("gompertz","exponential","gamma","genf","gengamma","weibull","weibullPH","loglogistic","lognormal")

SG_OS_fit<-fit.models(Surv(time,event)~1, data=SG_OS, distr = mods, method = "mle")

placebo_OS_fit<-fit.models(Surv(time,event)~1, data=SG_PFS, distr = mods, method = "mle")

SG_OS_fit$model.fitting

placebo_OS_fit$model.fitting # get AIC and BIC value of different parametric survival mdoels

#plot curves for all the parametric survival models

SG_OS_mle <- fit.models(formula = Surv(time, event) ~ 1, data = SG_OS, distr = mods, method = "mle")

plot(SG_OS_mle, xlab="Months", ylab=" Survival (%)")

placebo_OS_mle<- fit.models(formula = Surv(time, event) ~ 1, data = placebo_OS, distr = mods, method = "mle")

plot(placebo_OS_mle, xlab="Months", ylab=" Survival (%)")
